# A microRNA-based prediction model for lymph node metastasis in hepatocellular carcinoma

**DOI:** 10.18632/oncotarget.6534

**Published:** 2015-12-09

**Authors:** Li Zhang, Zuo-Lin Xiang, Zhao-Chong Zeng, Jia Fan, Zhao-You Tang, Xiao-Mei Zhao

**Affiliations:** ^1^ Department of Radiation Oncology, Zhongshan Hospital, Fudan University, Shanghai 200032, China; ^2^ Liver Cancer Institute, Zhongshan Hospital, Fudan University, Shanghai 200032, China

**Keywords:** hepatocellular carcinoma, lymph node metastasis, microRNA, prediction model, in situ hybridization

## Abstract

We developed an efficient microRNA (miRNA) model that could predict the risk of lymph node metastasis (LNM) in hepatocellular carcinoma (HCC). We first evaluated a training cohort of 192 HCC patients after hepatectomy and found five LNM associated predictive factors: vascular invasion, Barcelona Clinic Liver Cancer stage, miR-145, miR-31, and miR-92a. The five statistically independent factors were used to develop a predictive model. The predictive value of the miRNA-based model was confirmed in a validation cohort of 209 consecutive HCC patients. The prediction model was scored for LNM risk from 0 to 8. The cutoff value 4 was used to distinguish high-risk and low-risk groups. The model sensitivity and specificity was 69.6 and 80.2 %, respectively, during 5 years in the validation cohort. And the area under the curve (AUC) for the miRNA-based prognostic model was 0.860. The 5-year positive and negative predictive values of the model in the validation cohort were 30.3 and 95.5 %, respectively. Cox regression analysis revealed that the LNM hazard ratio of the high-risk versus low-risk groups was 11.751 (95 % CI, 5.110–27.021; *P* < 0.001) in the validation cohort. In conclusion, the miRNA-based model is reliable and accurate for the early prediction of LNM in patients with HCC.

## INTRODUCTION

Hepatocellular carcinoma (HCC) is the most frequent histological form of primary liver cancer [1] and the sixth most common cancer in the world [2]. HCC mortality ranks third among all cancers worldwide and causes 600,000 deaths per year [2]. Metastasis is the major risk factor for long-term survival of patients with post-hepatectomy HCC, and it contributes to the high recurrence rate of HCC [3, 4]. Lymph node metastasis (LNM) occurs in approximately 10.0 % of HCC patients during the follow-up period after hepatectomy [5], whereas the LNM rate is estimated as 33.8 % in HCC patients with extrahepatic metastasis [6]. Although LNM of HCC is reported to be sensitive to external beam radiotherapy [7], the prognosis of LNM patients is worse than that of patients without LNM [8]. Therefore, it is important to establish a predictive model and identify molecular markers for LNM to accurately assess the LNM risk in HCC patients. The factors involved in LNM of HCC are poorly documented, especially microRNAs (miRNAs).

MiRNAs are a class of small, non-coding, and 20-22 nucleotides long RNAs, which are important regulators of gene expression [9]. Recent studies report that specific miRNAs contribute to LNM in several cancers. MiR-31 promotes LNM in lung adenocarcinoma (ADC) [10]. Aberrant miRNAs, including high-expression miR-185-5p and miR-542-5p and low-expression miR-339-5p and miR-3923, contribute to LNM of breast cancer [11]. Yigit and coauthors confirmed that miR-10b targeting prevented and arrested LNM in breast cancer [12].

The miRNAs responsible for LNM in HCC are largely unknown. If miRNA expression profiling could be used to identify HCC patients at high risk for LNM, the high-risk population could receive prophylactic radiotherapy for regional lymph nodes. So, it might reduce LNM incidence and prolong overall survival time for patients. In this study, we established tissue microarrays (TMAs) and performed in situ hybridization (ISH) to discover novel and annotated miRNAs, which are associated with LNM in HCC patients. We identified significant miRNA signatures and developed a novel miRNA-based prediction model to identify HCC patients at high risk for LNM.

## RESULTS

### Expression of in situ hybridization biomarkers in tissue microarrays

**Figure 1:**
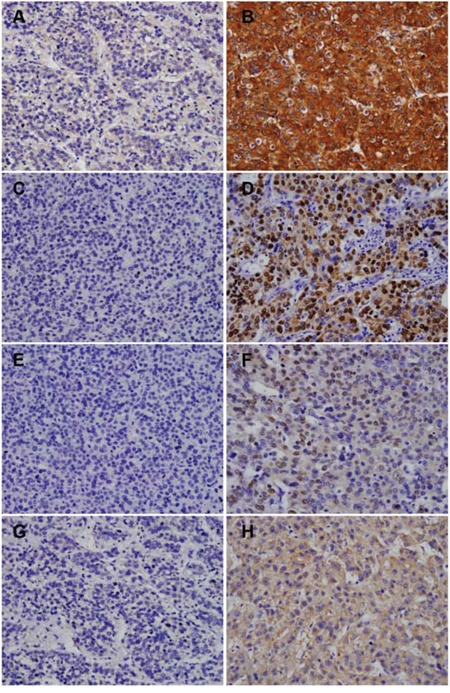
Expression of miR-145, miR-31, miR-92a, and miR-10b in hepatocellular carcinoma tissue microarrays Representative miR-145, miR-31, miR-92a, and miR-10b staining. Negative intratumoral expression of miR-145 **A.** positive intratumoral expression of miR-145 **B.** negative intratumoral expression of miR-31 **C.** positive intratumoral expression of miR-31 **D.** negative intratumoral expression of miR-92a **E.** positive intratumoral expression of miR-92a **F.** low intratumoral expression of miR-10b **G.** and high intratumoral expression of miR-10b **H.** Magnification × 200.

Figure 1 shows that miR-31 and miR-92a expression was identified in the cytoplasm and in the nucleus, whereas miR-145 and miR-10b expression was localized primarily to the cytoplasm of tumor cells. In the training cohort of 192 patients, positive miR-145 expression was detected in 47 patients (24.5 %), positive miR-31 expression was detected in 35 patients (18.2 %), positive miR-92a expression was detected in 55 patients (28.6 %), and high miR-10b expression was detected in 59 patients (30.7 %). In the validation cohort of 209 patients, positive miR-145 expression was detected in 54 patients (25.8 %), positive miR-31 expression was detected in 39 patients (18.7 %), positive miR-92a expression was detected in 66 patients (31.6 %), and high miR-10b expression was detected in 65 patients (31.1 %).

### Significant predictors of lymph node metastasis

Detailed characteristics of all patients analyzed in this study are presented in Table 1. The following 19 clinicopathological factors were analyzed in the training cohort: age, gender, HBsAg, hepatitis C virus antibody (HCV-Ab), alpha-fetoprotein (AFP), alanine aminotransferase (ALT), gamma-glutamyl transferase (γ-GT), liver cirrhosis, Child-Pugh score, tumor differentiation, tumor size, tumor number, tumor encapsulation, vascular invasion, Barcelona Clinic Liver Cancer (BCLC) stage, miR-145, miR-31, miR-92a, and miR-10b. Table 2 summarizes the association of these clinicopathological factors with the LNM status of HCC patients in the training cohort, which were determined by univariate analysis. These significant variables were adopted for multivariate analysis, which indicated that the following five independent variables could significantly predict the LNM risk in HCC: vascular invasion (*P* = 0.001), BCLC stage (*P* = 0.018), miR-145 (*P* = 0.014), miR-31 (*P* = 0.006), and miR-92a (*P* = 0.034) (Table 3). We also identified miR-145, miR-31 and miR-92a were correlated with vascular invasion and BCLC stage (Supplementary Tables S1 and S2).

**Table 1:** Clinicopathological characteristics of the study population

Variable	Training cohort				Validation cohort			
	All (*n* =192)	NLNM (*n* =169)	LNM (*n* =23)	*P*	All (*n* =209)	NLNM (*n* =186)	LNM (*n* =23)	*P*
Age								
≤ 51	94	82	12	0.742	102	88	14	0.220
>51	98	87	11		107	98	9	
Gender								
male	168	149	19	0.674	182	163	19	0.728
female	24	20	4		27	23	4	
HBsAg								
negative	53	46	7	0.746	54	49	5	0.634
positive	139	123	16		155	137	18	
HCV-Ab								
negative	188	165	23	0.457	205	182	23	0.479
positive	4	4	0		4	4	0	
AFP								
≤20	49	40	9	0.111	57	49	8	0.391
>20	143	129	14		152	137	15	
ALT								
≤40	112	100	12	0.523	121	108	13	0.888
>40	80	69	11		88	78	10	
γ-GT								
≤50	69	62	7	0.558	74	68	6	0.322
>50	123	107	16		135	118	17	
Liver cirrhosis								
no	31	27	4	1.000	33	28	5	0.599
yes	161	142	19		176	158	18	
Child-Pugh score								
A	190	167	23	0.601	207	184	23	0.618
B	2	2	0		2	2	0	
Tumor differentiation								
I–II	140	124	16	0.700	153	135	18	0.562
III–IV	52	45	7		56	51	5	
Tumor size, cm								
≤5	98	87	11	0.742	108	95	13	0.622
>5	94	82	12		101	91	10	
Tumor number								
single	139	123	16	0.746	153	138	15	0.359
multiple	53	46	7		56	48	8	
Tumor encapsulation								
complete	98	83	15	0.147	107	93	14	0.325
none	94	86	8		102	93	9	
Vascular invasion								
no	165	154	11	< 0.001*	164	152	12	0.003*
yes	27	15	12		45	34	11	
BCLC stage								
0-A	169	156	13	< 0.001*	177	166	11	< 0.001*
B-C	23	13	10		32	20	12	
MiR-145								
negative	145	138	7	< 0.001*	155	146	9	0.001*
positive	47	31	16		54	40	14	
MiR-31								
negative	157	147	10	< 0.001*	170	166	4	< 0.001*
positive	35	22	13		39	20	19	
MiR-92a								
negative	137	132	5	< 0.001*	143	141	2	< 0.001*
positive	55	37	18		66	45	21	
MiR-10b								
low expression	133	117	16	0.974	144	130	14	0.378
high expression	59	52	7		65	56	9	

HBsAg hepatitis B surface antigen, HCV-Ab hepatitis C virus antibody, AFP a-fetoprotein, ALT alanine aminotransferase, γ-GT γ-glutamyl transferase, BCLC stage Barcelona Clinic Liver Cancer stage,* significance values.

**Table 2:** Univariate analysis of clinicopathological factors associated with lymph node metastasis of hepatocellular carcinoma in the training cohort

Clinicopathological factors	Univariate		
	Hazard ratio	95% CI	*P*
Age			
≤ 51	1	0.402-3.479	0.761
>51	1.182		
Gender			
male	1	0.383-1.970	0.736
female	0.868		
HBsAg			
negative	1	0.344-2.047	0.701
positive	0.840		
HCV-Ab			
negative	1	0.000–1.094E7	0.757
positive	0.048		
AFP			
≤20	1	0.255-1.364	0.217
>20	0.590		
ALT			
≤40	1	0.637-3.286	0.337
>40	1.447		
γ-GT			
≤50	1	0.963-5.929	0.060
>50	2.389		
Liver cirrhosis			
no	1	0.281-2.438	0.731
yes	0.827		
Child-Pugh score			
A	1	0.000-4.640E4	0.666
B	0.048		
Tumor differentiation			
I–II	1	0.431-2.574	0.910
III–IV	1.053		
Tumor size, cm			
≤5	1	0.634-3.269	0.383
>5	1.440		
Tumor number			
single	1	0.458-2.710	0.811
multiple	1.115		
Tumor encapsulation			
complete	1	0.187-1.049	0.064
none	0.443		
Vascular invasion			
no	1	4.577-24.284	< 0.001*
yes	10.543		
BCLC stage			
0-A	1	1.050-6.765	0.039*
B-C	2.665		
MiR-145			
negative	1	1.123-5.777	0.025*
positive	2.547		
MiR-31			
negative	1	3.164-16.657	< 0.001*
positive	7.260		
MiR-92a			
negative	1	2.214-13.137	< 0.001*
positive	5.393		
MiR-10b			
low expression	1	0.334-1.981	0.650
high expression	0.814		

HBsAg hepatitis B surface antigen, HCV-Ab hepatitis C virus antibody, AFP a-fetoprotein, ALT alanine aminotransferase, γ-GT γ-glutamyl transferase, BCLC stage Barcelona Clinic Liver Cancer stage, CI, confidence interval. * significance values.

**Table 3:** Multivariate analysis of significant clinicopathological factors associated with lymph node metastasis of hepatocellular carcinoma in the training cohort

Clinicopathological factors	Multivariate			
	χ^2^ score	Hazard ratio	95% CI	*P*
Vascular invasion				
no		1	2.010-13.199	0.001*
yes	11.665	5.151		
BCLC stage				
0–A		1	1.204-6.984	0.018*
B–C	5.632	2.899		
MiR-145				
negative		1	1.295-10.176	0.014*
positive	6.011	3.630		
MiR-31				
negative		1	1.427-8.613	0.006*
positive	7.485	3.506		
MiR-92a				
negative		1	1.098-10.833	0.034*
positive	4.496	3.449		

Variables were adopted for their prognostic significance on univariate analysis.

BCLC stage Barcelona Clinic Liver Cancer stage, CI confidence interval, * significance values.

### Constructing the miRNA-based prediction model of lymph node metastasis in hepatocellular carcinoma

The miRNA-based prediction model was constructed as follows using multivariate analysis. Each chi-square (χ2) value was divided by the minimum multivariate analysis χ^2^ value of 4.496 to obtain a simple risk score for each significant variable according to its relative contribution to the multivariate analysis model (Table 4). The total risk score of every patient was the aggregate of all simple risk scores, ranging from 0 to 8 in both the training and the validation cohorts. The cutoff point of 4 for both cohorts was the best determinant to discriminate patient categories for low-risk and high-risk LNM groups using the χ^2^ test for linear trend.

**Table 4:** Components of the lymph node metastasis prediction score

Factor	Score
Vascular invasion	
no	0
yes	2.6
BCLC stage	
0-A	0
B-C	1.3
MiR-145	
negative	0
positive	1.3
MiR-31	
negative	0
positive	1.7
MiR-92a	
negative	0
positive	1

BCLC stage Barcelona Clinic Liver Cancer stage, Scores ≤4 indicates low risk and >4 indicates high risk.

Using the score of 4 as a cutoff point in the training cohort of 192 patients, 176 (91.7 %) and 16 (8.3 %) patients were in the low-risk and high-risk groups, respectively. In the low-risk and high-risk groups of the training cohort, 10/176 (5.7 %) and 13/16 (81.3 %) patients developed LNM (*P* < 0.05). The three-miRNA-based prognostic model sensitivity was 73.9 % and specificity was 79.4 % over 5 years and the area under the receiver operating characteristic (ROC) curve (AUC) was 0.906 (95 % CI, 0.842–0.969; *P* < 0.001), which indicates good reliability and validity (Supplementary Figure S1A). The 1- and 2-year cumulative LNM rates in the high-risk group were 78.1 and 85.4 %, respectively, whereas these values were 3.7 and 4.8 %, respectively, in the low-risk group. Patients in the high-risk group were found to have higher LNM rates (Supplementary Figure S1B). The 5-year positive and negative predictive values of the LNM prediction model were 32.8 and 95.7 %, respectively. Cox regression analysis determined that the LNM hazard ratio for high-risk versus low-risk groups was 32.071 (95 % CI, 12.599–81.636; *P* < 0.001).

### Real time qRT-PCR verification of miRNAs for LNM in HCC

Real time quantitative reverse transcriptase PCR(qRT-PCR) was performed to quantify miRNAs levels in formalin-fixed, paraffin-embedded (FFPE) specimens of 192 HCC patients. The following 19 clinicopathological factors analyzed in the training cohort were as follows: age, gender, HBsAg, hepatitis C virus antibody (HCV-Ab), AFP, ALT, γ-GT, liver cirrhosis, Child-Pugh score, tumor differentiation, tumor size, tumor number, tumor encapsulation, vascular invasion, BCLC stage, miR-145, miR-31, miR-92a, and miR-10b. Univariate analyses indicated that miR-145 (*P* = 0.036), miR-31 (*P* = 0.008), miR-92a (*P* = 0.025), vascular invasion (*P* < 0.001), and BCLC stage (*P* = 0.039) were associated with LNM in HCC patients. And miR-145 (*P* = 0.029), miR-31 (*P* = 0.018), miR-92a (*P* = 0.043), vascular invasion (*P* = 0.007), and BCLC stage (*P* = 0.024) were independent risk factors for LNM prediction in HCC patients by multivariate analyses.

### Validating the prognostic value of the miRNA-based model

The prognostic value of the miRNA-based model was validated using an independent validation cohort of 209 patients and the cutoff point of 4. The results indicated that 190/209 (90.9 %) and 19/209 (9.1 %) patients were categorized as low risk and high risk, respectively, in the validation cohort. In the low-risk and high-risk groups of the validation cohort, 11/190 (5.8 %) and 12/19 (63.2 %) patients developed LNM, respectively. Analysis of the validation cohort by ROC curve proved that the miRNA prediction model could predict LNM in HCC patients, with very high AUC of 0.860 (95 % CI, 0.773–0.948; *P* < 0.001) (Supplementary Figure S1C). The 5-year sensitivity and specificity of the prediction model were 69.6 and 80.2 %, respectively. The positive and negative values of the cutoff score for predicting LNM in HCC were 30.3 and 95.5 %, respectively, over 5 years.

Kaplan–Meier and log-rank tests were applied for analysis of time-to-LNM. The 1- and 2-year cumulative LNM rates in the high-risk category were 38.6 and 71.2 %, respectively, whereas these values were 4.5 and 11.7 %, respectively, in the low-risk category. Patients in the high-risk group had a higher risk for developing LNM than patients in the low-risk group (Supplementary Figure S1D). The Cox regression analysis hazard ratio for developing LNM of the high-risk versus low-risk groups was 11.751 (95 % CI, 5.110–27.021; *P* < 0.001).

## DISCUSSION

The incidence of LNM in extrahepatic metastases of HCC is 33.8 % [6], which contributes to the high mortality and poor prognosis of patients with HCC. However, an accurate model that can predict the LNM risk for patients with HCC has not been available. The LNM status depends primarily on LN biopsy, which may increase distant metastatic rates and reduce survival [21]. PET and CT analyses have low sensitivity for detecting tumor-positive small LNs <1 cm [22]. Therefore, the discovery of molecular markers to accurately assess the risk of LN involvement is of great importance. Aberrant forms of miRNAs have been reported in many cancer types, and considerable attention is focused on understanding the role of miRNAs in cancer development [23–25]. MiRNAs appear to have potential as molecular markers that can be developed to assess the LNM risk in many cancers [26–29]. In the current study, we sought to identify novel miRNAs that predict LNM in HCC.

We constructed and then validated a novel tool based on three miRNAs to predict LNM development for patients with HCC after hepatectomy. Our miRNA prediction model incorporates the following five factors: vascular invasion, BCLC stage, miR-145, miR-31, and miR-92a. Vascular invasion and BCLC stage are known prognostic factors for HCC [30, 31]. MiR-145 is known to promote LNM in colorectal cancer (CRC) [32]. The increased expression of heat shock protein 27 (Hsp-27), an up-regulated target of miR-145, is responsible for LNM in CRC [32]. MiR-31 promotes LNM in lung ADC through the activation of ERK1/2 signaling [10]. MiR-92a targeted suppressed E-Cadherin (CDH1) expression to promote LNM in esophageal squamous cell carcinoma [33]. And we are going to investigate the molecular mechanism of abnormal miRNAs expression associated with LNM in HCC by a large prospective research. It may have a little limitation for that some potential miRNAs may be not included in the study. We are going to screen significant miRNAs on a large scale through miRNA microarray in future research to enrich and perfect the microRNA-based prediction model for LNM in HCC.

To our knowledge, the significance of such a prediction model has not been unveiled in HCC, especially with respect to LNM. The miRNA-based prediction model effectively and accurately classifies post-hepatectomy HCC patients into groups with low risk and high risk of developing LNM. This model could be implemented for routine clinical use, and serve as a valuable tool for determining optimal treatment strategies for HCC patients. Traditional approaches include palliative radiotherapy after LNM. However, our model can identify high-risk HCC patients in advance, just after hepatectomy, which may help to guide the therapeutic strategy. These high-risk patients can be treated with prophylactic radiotherapy for regional lymph nodes during the earliest stages before LNM develops. This strategy could replace the current passive palliative treatment with active preventative treatment to suppress LNM development. Thus, the LNM incidence in HCC can be reduced, and the life quality and survival of patients can be improved. This predictive model also may provide new therapeutic targets and facilitate decisions regarding individualized clinical therapies.

In summary, the results confirm that our miRNA-based model is a good prognostic system to predict LNM in HCC patients. The predictive model is reliable and accurate for predicting LNM development in HCC patients. Further studies of larger cohorts are required, and the model should be validated in a prospective study. The mechanisms of abnormal miRNA expression in HCC are currently unclear, which requires investigation in future research.

## MATERIALS AND METHODS

### Ethics statement

Investigation had been approved by the Ethical Review Board of Zhongshan Hospital, Fudan University. Informed consent had been obtained from all participants included in this study.

### Patients and tissue specimens

All patients with HCC included in this study were diagnosed based on pathology. Patients with a history of other solid tumors were excluded from the study. None of the patients had distant metastasis before surgery, nor had they received anticancer therapy before surgery. All patients received chest radiography and abdominal ultrasonography examinations before surgery, and patients with extrahepatic metastasis were excluded from the study. Bone scanning was performed if bone pain was reported. If extrahepatic metastasis was suspected, computed tomography (CT) and/or magnetic resonance imaging (MRI) was performed to verify the occurrence of extrahepatic metastasis. Suitable FFPE tissue samples, complete clinicopathological examinations, and follow-up data were available for all patients. The tumor stage was defined in accordance with the BCLC staging system (2010 version). The histological grade of tumor differentiation was determined by the Edmondson grading system. Liver function was classified by the Child-Pugh scoring system. Tumor size depended on the maximum diameter of the tumor specimen. The extent of vascular invasion was identified by microscopic examination of the resected specimen.

The retrospective study was performed in two independent cohorts. The training cohort consisted of 192 consecutive HCC patients who underwent hepatectomy (all performed by the same surgical team) at the Liver Cancer Institute, Fudan University, from October 1999 to January 2006. The training cohort was used to develop a predictive model for LNM in HCC. The validation cohort consisted of 209 patients with HCC, who were recruited from August 2000 to May 2006. All patients in the validation cohort underwent hepatectomy by a different surgical team at the same institution. All patients in the training cohort were observed until December 2012, with a median follow-up time of 52.8 months (range 2.9-125.7 months). The follow-up cutoff time for the validation cohort was August 2012, with a median follow-up time of 53.7 months (range 3.8-128.6 months). During the follow-up period, 23 patients (12.0 %) in the training cohort and 23 patients (11.0 %) in the validation cohort developed LNM.

### Follow-up assessments

All patients received follow-up evaluations every 3 months after hepatectomy; each follow-up evaluation performed physical examinations and collected history documentation. Each follow-up examination included ultrasonographic examination of the liver and abdominal LNs; laboratory tests including liver function, AFP, ALT, and γ-GT; and evaluation of hematologic parameters. These examinations were performed by doctors who were blind to the study. Chest radiography was performed at 6-month intervals and a bone scan was performed annually. CT scanning or MRI was performed immediately when LNM was suspected. Metastatic LNs were indicated by one of the following: hypoechoic masses on ultrasonography, a central hypodensity region bordered by a faint hyperdensity rim on contrast CT scanning, or high signal intensity on T2-weighted MRI [5]. When a diagnosis of LNM was confirmed, the metastatic LNs received external beam radiotherapy [7]. Other recurrent foci were treated with radiotherapy, interventional therapy, or surgery.

### Tissue microarray construction and analysis

TMAs were constructed as described previously [13]. We constructed TMA slides (in collaboration with Biochip Company, Ltd., Shanghai, China) using HCC samples from 192 consecutive HCC patients in the training cohort and 209 consecutive HCC patients in the validation cohort. Slides stained with hematoxylin and eosin was screened to identify optimal intratumoral tumor tissue for analysis. Two cores with dimensions of 1.0 mm were punched from non-necrotic areas of tumor foci in the patient paraffin blocks. Sections (4-μm thickness) of the resulting TMA blocks were made using standard techniques.

### In situ hybridization analysis

ISH assays were performed with locked nucleic acid (LNA)-modified, 5′-digoxigenin (DIG)-labeled probes for mature human miRNAs (Exiqon, Vedbaek, Denmark). The following LNA primer sets were used: miR-145, 5′-AGGGATTCCTGGGAAAACTGGAC-3′; miR-31, 5′-AGCTATGCCAGCATCTTGCCT-3′; miR-92a, 5′-ACAGGCCGGGACAAGTGCAATA-3′; and miR-10b, 5′-CACAAATTCGGTTCTACAGGGTA-3′. Hybridization was performed using 4-μm sections of FFPE tissue. Briefly, slides were dewaxed, rehydrated, and acetylated with 0.25 % acetic anhydride. Then, sections were prehybridized in hybridization solution (Exiqon, Vedbaek, Denmark) for 1 h at 55°C. Hybridization was performed with 20 μl of a diluted solution (1:500) of the corresponding LNA probes overnight at 56°C. Slides were then washed once with 5 × SSC buffer followed by two washes with 0.2 × SSC buffer at 60°C. Chromogenic detection of signals was performed using an anti-DIG antibody (Zytovision, Germany) at 37°C and alkaline phosphatase-conjugated secondary antibody (Zytovision, Germany) according to the manufacturer's instructions.

### Evaluation methods for determining miRNA expression

Tissue sections were blindly examined by two experienced pathologists using an Olympus BX51 microscope (Olympus BX51, CCD: DP71, Japan). The average of the two scores was calculated. The chromogenic intensity of miR-145 expression was visually semi-quantified as follows: 0 = no signal, 1 = weak signal, 2 = intermediate signal, and 3 = strong signal. The percentage of the visually determined signal intensity was expressed as follows: 0, 0 %; 1, <30 %; and 2, >30 %. The intensity and percentage scores were added to give a final score of 0–5. Expression was determined based on the final score as follows: negative expression, final score ≤ 4; positive expression, 4 < final score ≤ 5 [14]. MiR-31 expression levels were scored using a previously described ISH scoring system, which combines the signal intensity (0 = no signal, 1 = weak signal, 2 = intermediate signal, and 3 = strong signal) and percentage of positive cells (0, 0 %; 1, <30 %; and 2, >30 %) to produce a score that ranges from 0–5. Expression levels were characterized as negative, final score ≤3 or positive, 3 < final score ≤ 5 [15]. MiR-92a expression levels were scored using the following four-tier scoring system according to the staining intensity: 0 = negative, 1 = weak positive, 2 = moderate and 3 = strong positive. Expression levels were considered as negative, final score ≤ 0.5 or positive 0.5 < final score ≤ 3 [16]. Cytoplasmic miR-10b staining was categorized as follows: low-expression, < 20 % of cells stained; high-expression, ≥ 20 % of cells stained [17].

### Extraction of miRNAs from FFPE tissue and validation by real time qRT-PCR

FFPE specimens stained with hematoxylin and eosin were identified optimal intratumoral tissue of 192 hepatocellular carcinoma (HCC) patients. Total RNA including miRNA was extracted from FFPE samples using miRNeasy FFPE kit (Qiagen) according to manufacturer's instructions. Briefly, the samples were deparaffinsed and lysed with proteinase K digestion. After a short incubation at a higher temperature, DNase treatment was performed to eliminate genomic DNA, including very small DNA fragments which were often present in FFPE samples after long-term fixation and storage. Followed by the addition of buffer RBC and 100% ethanol, then the sample was applied to RNeasy MinElute spin columns. Finally, samples were washed and eluted in RNase free water. RNA concentrations were measured via absorbance spectrophotometry on a NanoVue Plus instrument (GE, General Electric Company).

The expression levels of the candidate miRNAs were validated by real time qRT-PCR. Primers for U6, miR-145, miR-31, miR-92a, and miR-10b were purchased from GeneCopoeia. Real time qRT-PCR was performed by the SYBR Green PCR method using All-in-One™ miR qRT-PCR detection kit (GeneCopoeia, China) with miRNA specific primers. All samples were run in triplicate, and the 2^−ΔΔCt^ method was used to analyse the miRNA expression levels. The qRT-PCR was performed on a 7500 Real-Time PCR System (Applied Biosystems). U6 snRNA was used as the endogenous control.

### Statistical analysis

Correlations between clinicopathological features and LNM status were analyzed by Pearson's χ^2^ test and Fisher's exact test. Then, univariate analysis and multivariate analysis were performed to determine statistically significant variables and devise a simple risk score, which was the corresponding estimated coefficient divided by the minimum χ^2^ value [18]. The final score of every patient was the aggregate of all risk scores. The optimal cutoff point was determined by the χ^2^ test for linear trend for the best separation of LNM risk [19, 20]. Prediction performance of the miRNA-based model was evaluated using AUC. The temporal interval from the date of surgery to the date of LNM incidence was considered as time-to-LNM, which was analyzed by Kaplan-Meier and log-rank tests. Cox regression analysis was used to estimate relative LNM risk of the high-risk versus low-risk groups. Two-sided *P* values < 0.05 were considered as statistically significant. All statistical analyses were conducted with SPSS version 20.0 (SPSS IBM).

## SUPPLEMENTARY FIGURE AND TABLES



## References

[1] el-Serag HB (2001). Epidemiology of hepatocellular carcinoma. Clin Liver Dis.

[2] Torre LA, Bray F, Siegel RL, Ferlay J, Lortet-Tieulent J, Jemal A (2015). Global cancer statistics, 2012. CA Cancer J Clin.

[3] Huang W, Chen Z, Shang X, Tian D, Wang D, Wu K, Fan D, Xia L (2015). Sox12, a direct target of FoxQ1, promotes hepatocellular carcinoma metastasis through up-regulating Twist1 and FGFBP1. Hepatology.

[4] El-Serag HB (2011). Hepatocellular carcinoma. N Engl J Med.

[5] Xiang ZL, Zeng ZC, Fan J, Tang ZY, Zeng HY, Gao DM (2011). Gene expression profiling of fixed tissues identified hypoxia-inducible factor-1α, VEGF, and matrix metalloproteinase-2 as biomarkers of lymph node metastasis in hepatocellular carcinoma. Clin Cancer Res.

[6] Natsuizaka M, Omura T, Akaike T, Kuwata Y, Yamazaki K, Sato T, Karino Y, Toyota J, Suga T, Asaka M (2005). Clinical features of hepatocellular carcinoma with extrahepatic metastases. J Gastroenterol Hepatol.

[7] Zeng ZC, Tang ZY, Fan J, Qin LX, Ye SL, Zhou J, Sun HC, Wang BL, Wang JH (2005). Consideration of role of radiotherapy for lymph node metastases in patients with HCC: retrospective analysis for prognostic factors from 125 patients. Int J Radiat Oncol Biol Phys.

[8] Amini N, Ejaz A, Spolverato G, Maithel SK, Kim Y, Pawlik TM (2014). Management of lymph nodes during resection of hepatocellular carcinoma and intrahepatic cholangiocarcinoma: a systematic review. J Gastrointest Surg.

[9] Calin GA, Croce CM (2006). MicroRNA signatures in human cancers. Nat Rev Cancer.

[10] Meng W, Ye Z, Cui R, Perry J, Dedousi-Huebner V, Huebner A, Wang Y, Li B, Volinia S, Nakanishi H, Kim T, Suh SS, Ayers LW, Ross P, Croce CM, Chakravarti A, Jin VX, Lautenschlaeger T (2013). MicroRNA-31 predicts the presence of lymph node metastases and survival in patients with lung adenocarcinoma. Clin Cancer Res.

[11] Wang B, Li J, Sun M, Sun L, Zhang X (2014). miRNA expression in breast cancer varies with lymph node metastasis and other clinicopathologic features. IUBMB Life.

[12] Yigit MV, Ghosh SK, Kumar M, Petkova V, Kavishwar A, Moore A, Medarova Z (2013). Context-dependent differences in miR-10b breast oncogenesis can be targeted for the prevention and arrest of lymph node metastasis. Oncogene.

[13] Xiang ZL, Zeng ZC, Tang ZY, Fan J, Sun HC, Tan YS (2011). Expression of cytokeratin 19 and matrix metalloproteinase 2 predicts lymph node metastasis in hepatocellular carcinoma. Mol Biol Rep.

[14] Sempere LF, Christensen M, Silahtaroglu A, Bak M, Heath CV, Schwartz G, Wells W, Kauppinen S, Cole CN (2007). Altered MicroRNA expression confined to specific epithelial cell subpopulations in breast cancer. Cancer Res.

[15] Liu X, Sempere LF, Ouyang H, Memoli VA, Andrew AS, Luo Y, Demidenko E, Korc M, Shi W, Preis M, Dragnev KH, Li H, Direnzo J, Bak M, Freemantle SJ, Kauppinen S, Dmitrovsky E (2010). MicroRNA-31 functions as an oncogenic microRNA in mouse and human lung cancer cells by repressing specific tumor suppressors. J Clin Invest.

[16] Lao IW, Cui F, Zhu H (2015). Quantitation of microRNA-92a in colorectal adenocarcinoma and its precancerous lesions: Co-utilization of in situ hybridization and spectral imaging. Oncol Lett.

[17] Santarpia L, Calin GA, Adam L, Ye L, Fusco A, Giunti S, Thaller C, Paladini L, Zhang X, Jimenez C, Trimarchi F, El-Naggar AK, Gagel RF (2013). A miRNA signature associated with human metastatic medullary thyroid carcinoma. Endocr Relat Cancer.

[18] Wong VW, Chan SL, Mo F, Chan TC, Loong HH, Wong GL, Lui YY, Chan AT, Sung JJ, Yeo W, Chan HL, Mok TS (2010). Clinical scoring system to predict hepatocellular carcinoma in chronic hepatitis B carriers. J Clin Oncol.

[19] Feinstein AR (1972). Clinical biostatistics. XV. The process of prognostic stratification. I. Clin Pharmacol Ther.

[20] Ueno S, Tanabe G, Sako K, Hiwaki T, Hokotate H, Fukukura Y, Baba Y, Imamura Y, Aikou T (2001). Discrimination value of the new western prognostic system (CLIP score) for hepatocellular carcinoma in 662 Japanese patients. Cancer of the Liver Italian Program. Hepatology.

[21] McGuirt WF, McCabe BF (1978). Significance of node biopsy before definitive treatment of cervical metastatic carcinoma. Laryngoscope.

[22] Al-Sarraf N, Gately K, Lucey J, Wilson L, McGovern E, Young V (2008). Lymph node staging by means of positron emission tomography is less accurate in non-small cell lung cancer patients with enlarged lymph nodes: analysis of 1,145 lymph nodes. Lung Cancer.

[23] Sato F, Hatano E, Kitamura K, Myomoto A, Fujiwara T, Takizawa S, Tsuchiya S, Tsujimoto G, Uemoto S, Shimizu K (2011). MicroRNA profile predicts recurrence after resection in patients with hepatocellular carcinoma within the Milan Criteria. PLoS One.

[24] Zhang JX, Song W, Chen ZH, Wei JH, Liao YJ, Lei J, Hu M, Chen GZ, Liao B, Lu J, Zhao HW, Chen W, He YL, Wang HY, Xie D, Luo JH (2013). Prognostic and predictive value of a microRNA signature in stage II colon cancer: a microRNA expression analysis. Lancet Oncol.

[25] Fisher JN, Terao M, Fratelli M, Kurosaki M, Paroni G, Zanetti A, Gianni M, Bolis M, Lupi M, Tsykin A, Goodall GJ, Garattini E (2015). MicroRNA networks regulated by all-trans retinoic acid and Lapatinib control the growth, survival and motility of breast cancer cells. Oncotarget.

[26] Chen Y, Min L, Zhang X, Hu S, Wang B, Liu W, Wang R, Gu X, Shen W, Lv H, Zou J, Chen Y, Xu X, Chen L (2013). Decreased miRNA-148a is associated with lymph node metastasis and poor clinical outcomes and functions as a suppressor of tumor metastasis in non-small cell lung cancer. Oncol Rep.

[27] Chu HW, Cheng CW, Chou WC, Hu LY, Wang HW, Hsiung CN, Hsu HM, Wu PE, Hou MF, Shen CY, Yu JC (2014). A novel estrogen receptor-microRNA 190a-PAR-1-pathway regulates breast cancer progression, a finding initially suggested by genome-wide analysis of loci associated with lymph-node metastasis. Hum Mol Genet.

[28] Huang B, Li H, Huang L, Luo C, Zhang Y (2015). Clinical significance of microRNA 138 and cyclin D3 in hepatocellular carcinoma. J Surg Res.

[29] Chen P, Zhao X, Ma L (2013). Downregulation of microRNA-100 correlates with tumor progression and poor prognosis in hepatocellular carcinoma. Mol Cell Biochem.

[30] Zhu XD, Zhang JB, Zhuang PY, Zhu HG, Zhang W, Xiong YQ, Wu WZ, Wang L, Tang ZY, Sun HC (2008). High expression of macrophage colony-stimulating factor in peritumoral liver tissue is associated with poor survival after curative resection of hepatocellular carcinoma. J Clin Oncol.

[31] Park SK, Jung YK, Chung DH, Kim KK, Park YH, Lee JN, Kwon OS, Kim YS, Choi DJ, Kim JH (2013). Factors influencing hepatocellular carcinoma prognosis after hepatectomy: a single-center experience. Korean J Intern Med.

[32] Yuan W, Sui C, Liu Q, Tang W, An H, Ma J (2014). Up-regulation of microRNA-145 associates with lymph node metastasis in colorectal cancer. PLoS One.

[33] Chen ZL, Zhao XH, Wang JW, Li BZ, Wang Z, Sun J, Tan FW, Ding DP, Xu XH, Zhou F, Tan XG, Hang J, Shi SS, Feng XL, He J (2011). microRNA-92a promotes lymph node metastasis of human esophageal squamous cell carcinoma via E-cadherin. J Biol Chem.

