# Supplementary material for: A microRNA-based prediction model for lymph node metastasis in hepatocellular carcinoma
Source: Oncotarget. 2015 Dec 9;7(3):3587–98. doi: 10.18632/oncotarget.6534 (PMC4823129; doi:10.18632/oncotarget.6534)
Supplement: Supplementary file 1 [file oncotarget-07-3587-s001.pdf]

SUPPLEMENTARY FIGURE AND TABLES

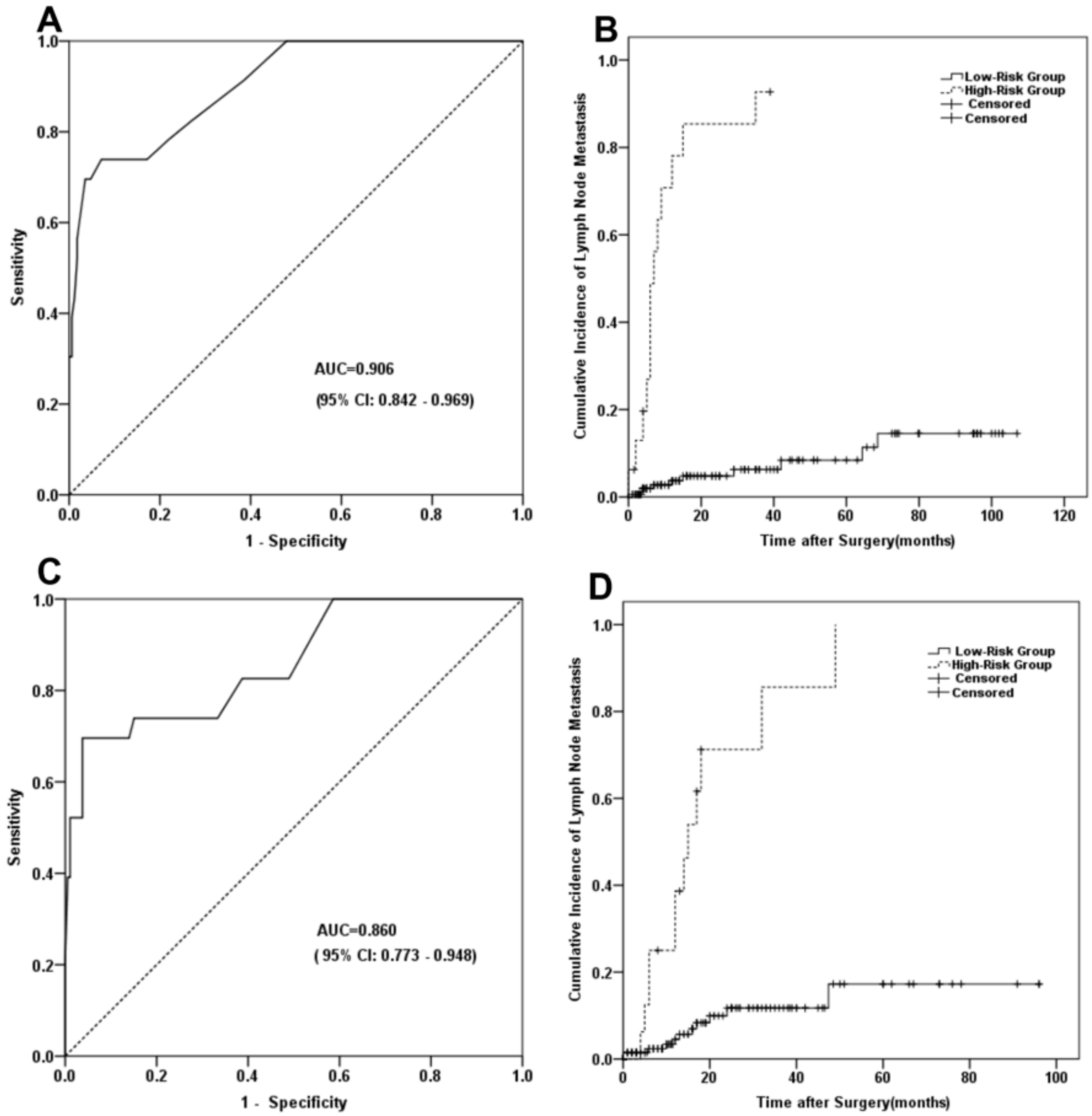

Supplementary Figure S1: A. ROC analysis of the miRNA-based model for the risk of LNM in HCC patients in the training cohort. **B.** Kaplan-Meier and log-rank analysis of cumulative incidence of LNM and low- and high-risk groups in HCC patients in the training cohort **C.** ROC analysis of the miRNA-based model for the risk of LNM in HCC patients in the validation cohort **D.** Kaplan-Meier and log-rank analysis of cumulative incidence of LNM and low- and high-risk groups in HCC patients in the validation cohort.

Supplementary Table S1: The correlations between miRNAs expression and vascular invasion in two cohorts

Variable	Vascular invasion of Training cohort				Vascular invasion of Validation cohort			
	All (*n* = 192)	no (*n* = 165)	yes (*n* = 27)	*P*	All (*n* = 209)	no (*n* = 164)	yes (*n* = 45)	*P*
MiR-145								
negative	145	130	15	0.009*	155	116	39	0.031*
positive	47	35	12		54	48	6	
MiR-31								
negative	157	139	18	0.028*	170	139	31	0.016*
positive	35	26	9		39	25	14	
MiR-92a								
negative	137	124	13	0.004*	143	106	37	0.025*
positive	55	41	14		66	58	8	
MiR-10b								
low expression	133	114	19	0.894	144	109	35	0.146
high expression	59	51	8		65	55	10	

*significance values.

Supplementary Table S2: The correlations between miRNAs expression and BCLC stage in two cohorts

Variable	BCLC stage of Training cohort				BCLC stage of Validation cohort			
	**All (n = 192)**	**0-A (n = 169)**	**B-C (n = 23)**	***P***	**All (n = 209)**	**0-A (n = 177)**	**B-C (n = 32)**	***P***
MiR-145								
negative	145	133	12	0.006*	155	136	19	0.038*
positive	47	36	11		54	41	13	
MiR-31								
negative	157	143	14	0.006*	170	152	18	< 0.001*
positive	35	26	9		39	25	14	
MiR-92a								
negative	137	125	12	0.030*	143	126	17	0.043*
positive	55	44	11		66	51	15	
MiR-10b								
low expression	133	117	16	0.974	144	122	22	0.984
high expression	59	52	7		65	55	10	

BCLC stage: Barcelona Clinic Liver Cancer stage.
*significance values.
